# Extracellular loops of the serotonin transporter act as a selectivity filter for drug binding

**DOI:** 10.1016/j.jbc.2021.100863

**Published:** 2021-06-09

**Authors:** Eray Esendir, Verena Burtscher, Jonathan A. Coleman, Rong Zhu, Eric Gouaux, Michael Freissmuth, Walter Sandtner

**Affiliations:** 1 Institute of Pharmacology and the Gaston H. Glock Research Laboratories for Exploratory Drug Development, Center of Physiology and Pharmacology, Medical University of Vienna, Austria; 2 Vollum Institute, Oregon Health & Science University, Portland, Oregon, USA; 3 Institute of Biophysics, Johannes Kepler University Linz, Austria; 4 Howard Hughes Medical Institute, Oregon Health & Science University, Portland, Oregon, USA

**Keywords:** serotonin transporter, selective serotonin reuptake inhibitor (SSRI), kinetics, membrane transport, electrophysiology, protein structure, EL, extracellular loops, DMEM, Dulbecco's modified Eagle's medium, FBS, fetal bovine serum, PBS, phosphate-buffered saline, PMSF, phenylmethylsulfonyl fluoride, SERT, serotonin transporter, TM, transmembrane

## Abstract

The serotonin transporter (SERT) shapes serotonergic neurotransmission by retrieving its eponymous substrate from the synaptic cleft. Ligands that discriminate between SERT and its close relative, the dopamine transporter DAT, differ in their association rate constant rather than their dissociation rate. The structural basis for this phenomenon is not known. Here we examined the hypothesis that the extracellular loops 2 (EL2) and 4 (EL4) limit access to the ligand-binding site of SERT. We employed an antibody directed against EL4 (residues 388–400) and the antibody fragments 8B6 scFv (directed against EL2 and EL4) and 15B8 Fab (directed against EL2) and analyzed their effects on the transport cycle of and inhibitor binding to SERT. Electrophysiological recordings showed that the EL4 antibody and 8B6 scFv impeded the initial substrate-induced transition from the outward to the inward-facing conformation but not the forward cycling mode of SERT. In contrast, binding of radiolabeled inhibitors to SERT was enhanced by either EL4- or EL2-directed antibodies. We confirmed this observation by determining the association and dissociation rate of the DAT-selective inhibitor methylphenidate *via* electrophysiological recordings; occupancy of EL2 with 15B8 Fab enhanced the affinity of SERT for methylphenidate by accelerating its binding. Based on these observations, we conclude that (i) EL4 undergoes a major movement during the transition from the outward to the inward-facing state, and (ii) EL2 and EL4 limit access of inhibitors to the binding of SERT, thus acting as a selectivity filter. This insight has repercussions for drug development.

Within the solute carrier-6 family, the closely related monoamine transporters for serotonin (SERT/SLC6A4), dopamine (DAT/SLC6A3), and norepinephrine (NET/SLC6A2) form a separate branch (1). Monoamine transporters, and in particular SERT, are arguably the most studied and hence best understood solute carriers (2). In fact, the structure of SERT is known in atomic detail in three conformations, which are visited during the transport cycle, *i.e.*, the outward, the occluded, and the inward-facing state (3, 4). SERT is a secondary active transporter, which harvests the electrochemical Na^+^ gradient as a driving force for substrate translocation across the bilayer: it must bind cosubstrate ions, *i.e.*, two sodium and one chloride ion. The kinetics of the transport cycle have been studied in real time, which allowed for defining the rate-limiting step in the conformational transition from the substrate-free inward-facing to outward-facing state (5), the sequence of cosubstrate ion binding and release (6), and the factors that promote the switch from the forward transport mode to the exchange mode (7). SERT is a popular target for both drugs of therapeutic relevance (*e.g.*, antidepressants) and illicit amphetamine-like compounds (8). Thus studies on SERT are not only important to inform drug design and to understand its role in serotonergic neurotransmission and neuromodulation but also to gauge the significance of SERT in drug abuse and in psychiatric disorders. In addition, the insights from these studies also shape concepts of the transport cycle of other secondary active transporters (9) and of diseases caused by mutations in *slc6* transporter-encoding genes that affect folding of the expressed protein (10).

SERT harbors two binding sites, a central binding site (S1) and a vestibular binding site (S2 or allosteric site) (11). The central binding site (S1) accommodates substrate and cosubstrate ions and inhibitors and lies within the hydrophobic core, which is formed by 12 transmembrane (TM) helices (3, 12, 13). The first ten helices are organized as an inverted repeat, where TM1 to TM5 and TM6 to TM10 are arranged in a pseudosymmetric fashion; this tertiary structure is referred to as the LeuT fold (14). Ligands access S1 *via* an entry pathway, which harbors the second vestibular-binding site (3, 15, 16, 17). Ligand selectivity for SERT *versus* the closely related DAT is determined by the association rather than the dissociation rate constant (18). This observation indicates that access to the binding pocket is, at least in part, rate-limiting. SERT has two large extracellular loops, EL2 and EL4, connecting TM3 and TM4 and TM7 and TM8, respectively. EL4 is comprised of two helical portions and has a wedge-like shape. The positions of EL4 differ substantially in the outward and the inward-facing states of LeuT: in the inward-facing state, EL4 shields the substrate-binding site of LeuT (19). The apparent movement of EL4 is more subtle in the inward-facing state of SERT (4). However, a mutation in EL4 (L406E), which is located in the vicinity of the tip of the wedge, enhances the binding of inhibitors and reduces substrate turnover rate (20). This is consistent with a role of EL4 in controlling access to the central binding site. Circumstantial evidence indicates that EL2 is important for substrate translocation rather than for ligand binding (21).

Here we analyzed the effects of antibody fragments directed against EL2 and EL4 and of an EL4-antibody on the substrate-driven conformational cycle of SERT and examined the hypothesis that EL4 and EL2 limited ligand access to the central binding site. Our observations show that steric hindrance in the movement of EL4—but not of EL2—impeded the initial conformational transition required for substrate translocation. In contrast, occupancy by antibody or Fabs directed against EL2 and EL4 enhanced inhibitor binding to SERT by promoting the association without affecting the dissociation, a finding consistent with a role of EL2 and EL4 in contributing to a selectivity filter in SERT. This conjecture was reinforced by using the DAT-selective inhibitor methylphenidate.

## Results

### Inhibition by 8B6 scFv—but not by 15B8 Fab—of the initial, substrate-induced conformational change of SERT

**Figure 1:**
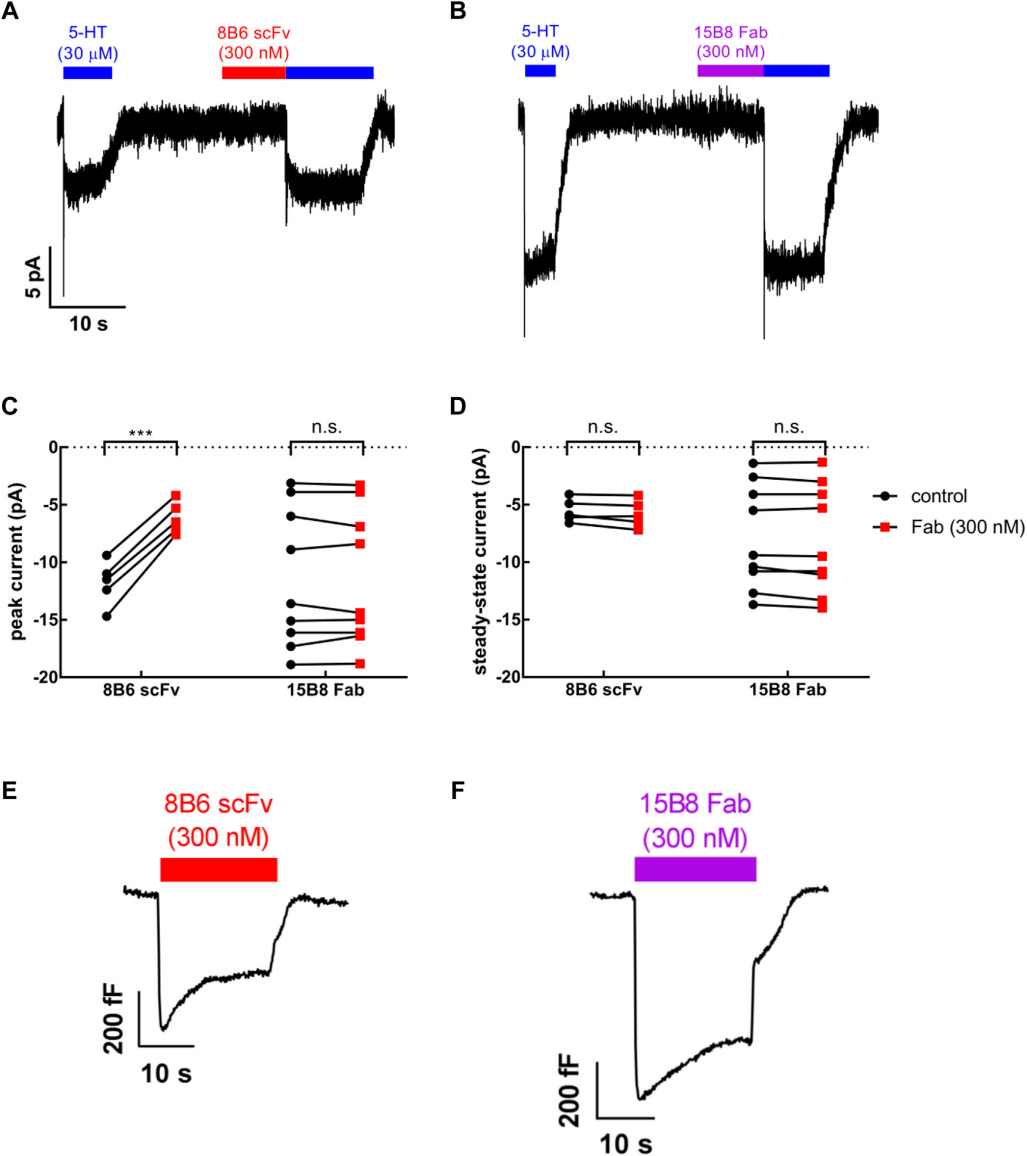
**Whole-cell patch-clamp recordings of currents associated with 5-HT transport (*A–D*) and membrane capacitance changes (*E*, *F*) in the presence of 8B6 scFv (*A*, C, *E*) and of 15B8 Fab (*B*, *D*, *F*).***A* and *B,* representative traces elicited by rapid application of 5-HT (30 μM) onto HEK293 stably expressing GFP-tagged SERT before and after superfusion with (*A*) 8B6 scFv (300 nM) or (*B*) 15B8 Fab (300 nM) in physiological ion gradients at a holding potential of –60 mV. *C*, comparison of the peak current amplitudes before and after 8B6 scFv (mean ± SD: –11.8 ± 2.0 and –6.2 ± 2.0 pA, respectively, n = 5, *p* = 0.0001; paired *t* test) or 15B8 Fab (–11.0 ± 3.6 and –11.6 ± 3.6 pA, respectively, n = 9, not significant, *p* = 0.86; paired *t* test) binding to SERT. *D*, comparison of the steady-state current amplitudes before and after application of 8B6 scFv (mean ± SD: –5.5 ± 1.0 and –5.8 ± 1.2 pA, respectively, n = 5, *p* = 0.11; paired *t* test) or 15B8 Fab (–7.8 ± 4.5 and –8.0 ± 4.7 pA, respectively, n = 9, not significant, *p* = 0.09; paired *t* test). *E* and *F,* representative traces of the apparent reduction in the membrane capacitance of HEK293 cells stably expressing GFP-tagged SERT after superfusion with 8B6 scFv (300 nM, *E*) or 15B8 Fab (300 nM, *F*).

The transport cycle of SERT can be studied by electrophysiological recordings. Serotonin (5-hydroxytryptamine, 5-HT) transport induces a current through SERT with two components, a peak component followed by a steady-state component (Fig. 1, *A* and *B*). The peak component corresponds to the initial conformational change on SERT, induced by the binding and translocation of the substrate and cosubstrates. It is inwardly directed due to the movement of net positive charges (Na^+^ and the ionized substrate) through the membrane electric field (5) as SERT undergoes conformational changes from outward-open to occluded and inward-open states upon substrate binding and release (4). SERT releases substrate in the inward-open state, and K^+^ is bound to the transporter during the return step to complete the transport cycle. This cycle continues as long as the substrate is applied from the extracellular side. The cycling of the transporter creates a Na^+^ conductance, which gives rise to the second current component, *i.e.*, the steady-state current (5).

The 15B8 Fab and 8B6 scFv are directed against an epitope in extracellular loop 2 (EL2) and epitopes in EL2 and EL4, respectively (3, 4). It was previously reported that binding of both 8B6 scFv and 15B8 Fab to SERT completely blocked substrate uptake; in contrast, binding of 15B8 Fab alone did not inhibit substrate translocation (4). We examined the ability of these two antibody fragments on the transport cycle in real time by whole-cell patch-clamp recordings. The experimental protocol (see schematic representation in Fig. 1, *A* and *B*) relied on the application of 30 μM 5-HT for 5 s to elicit a reference current. This was followed by a washout for 20 s, which allowed for the decay of the current to baseline. Subsequently, the cell was superfused with a saturating concentration (300 nM) of 8B6 scFv (Fig. 1*A*) or of 15B8 Fab (Fig. 1*B*) to allow for binding of the antibodies to SERT. When 8B6 scFv-bound SERT was challenged by reapplication of the substrate, the peak current was reduced by about 50%, but the steady-state current was not altered (Fig. 1, *A* and *C*). In contrast, in the 15B8 Fab-bound complex, neither the peak nor the steady-state currents were affected (Fig. 1, *B* and *D*).

Because 15B8 Fab did not elicit any change in the substrate-induced currents, we used an independent approach to verify that 15B8 Fab could bind to the transporter under the conditions employed in our electrophysiological recordings: we relied on the observation that binding of a charged molecule to the membrane or to a membrane protein neutralizes surface charge and can be measured by following the change in membrane capacitance (22). HEK293 cells stably expressing SERT were superfused with 15B8 Fab (300 nM) or—as a control—8B6 scFv (300 nM) and the changes in membrane capacitance were recorded. Both 8B6 scFv (Fig. 1*E*) and 15B8 Fab (Fig. 1*F*) caused a drop in membrane capacitance (by about 400 fF and 600 fF, respectively), which was reversed upon removal of the antibody fragments. We therefore conclude that 15B8 Fab can bind to SERT but does not affect the substrate-induced current.

**Figure 2:**
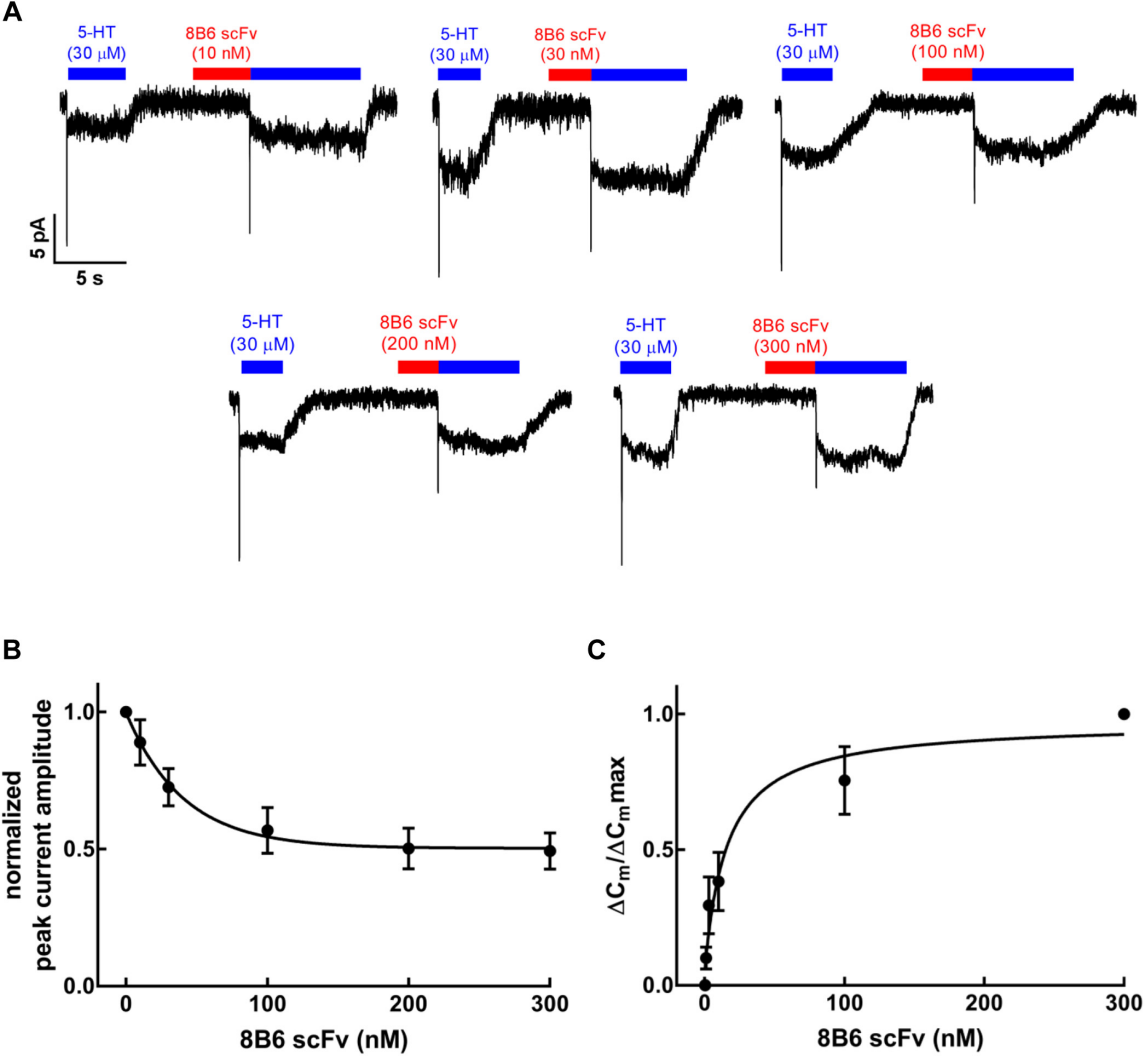
**Concentration-dependent inhibition of the peak currents through SERT by 8B6 scFv.***A*, representative traces depict currents induced by 30 μM 5-HT before and after superfusion for 5 s with the indicated concentrations of 8B6 scFv. *B*, concentration–response curve for 8B6 scFv-dependent reduction in peak current, which was normalized to the peak current amplitude recorded in the absence of 8B6 scFv (n = 4 for each concentration; error bars indicate SD). The solid curve was drawn by fitting the data to the equation for a monophasic inhibition to a residual basal level resulting in an estimated IC_50_ = 27.9 ± 7.3 nM (mean ± SD). *C*, concentration–response curve for the 8B6 scFv induced change (n = 4 for each concentration; error bars indicate SD), normalized to the maximum change in the membrane capacitance upon 8B6 scFv infusion. The data were fitted to the equation for a rectangular hyperbola resulting in an EC_50_ estimate of 15.7 ± 5.1 (mean ± SD).

Next, we determined the concentration-dependent inhibition of the 5-HT-induced peak current by 8B6 scFv. As shown in Figure 2*A*, the peak current amplitude progressively decreased with increasing concentrations of 8B6 scFv. We normalized the peak current recorded after superfusion with 8B6 scFv to the reference peak current elicited prior to application of 8B6 scFv in order to account for intercell differences and plotted these normalized current amplitudes as a function of 8B6 scFv concentration. This resulted in a monophasic inhibition curve with a plateau at about 50%; half-maximum inhibition was seen at 27.9 ± 7.3 nM (mean ± SD). We also independently estimated the affinity of 8B6 scFv by recording the concentration-dependent reduction in membrane capacitance (ΔCm). Variations in cell size and hence in membrane capacitance were accounted for by normalizing ΔC_m_ to the maximum drop in membrane capacitance (ΔC_m_max). The relation of 8B6 scFv concentration to the change in membrane capacitance (ΔCm/ΔC_m_max) was adequately described by a rectangular hyperbola, yielding an EC_50_ estimate of 15.7 ± 5.1 nM (mean ± SD). Taken together, these observations indicate that 8B6 scFv—but not 15B8 Fab—impedes the conformational change of SERT, which is triggered by the binding of serotonin. However, once serotonin has induced transport, the transporter may be relieved from the inhibitory action of 8B6 scFv, allowing it to cycle effectively through its conformational transitions to support the steady-state current. In contrast, sole occupancy of EL2 by 15B8 Fab neither impedes entry into nor progression through the transport cycle.

### EL4-directed antibody recapitulates the effect of 8B6 scFv on the transport cycle

**Figure 3:**
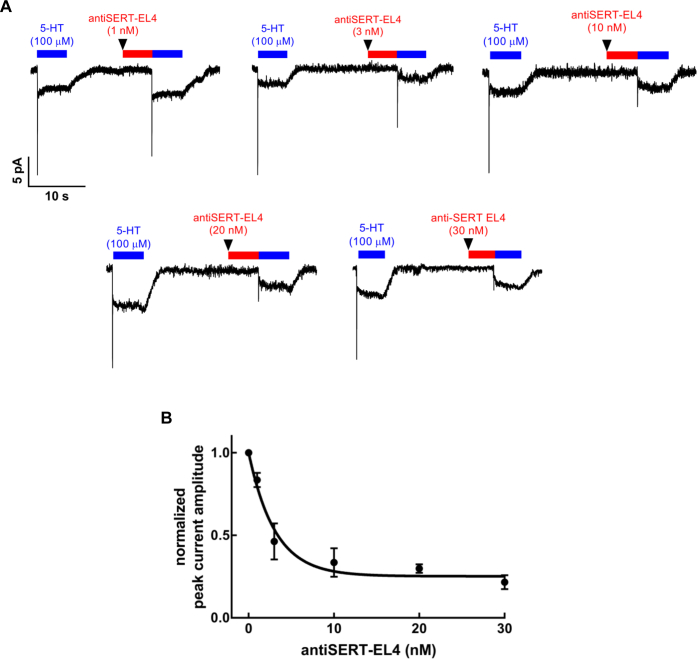
**Concentration-dependent inhibition of the substrate-induced peak current inhibition by the antiSERT-EL4 antibody.***A*, representative current traces elicited by 5-HT (100 μM) before and after superfusion with 1, 3, 10, 20, or 30 nM of antiSERT-EL4. *B,* concentration–response curve for antiSERT-EL4 antibody-mediated reduction in peak current (n = 8, 4, 4, 7, and 12, respectively; error bars are SD). The solid curve was drawn by fitting the data to the equation for a monophasic inhibition to a residual basal level resulting in an estimated IC_50_ = 2.2 ± 0.4 nM (mean ± SD).

Because 8B6 scFv binds to both EL2 and EL4 (3), it was not clear if its action was due to binding to EL2, to EL4, or the combination of both. Accordingly, we used an affinity-purified antibody raised against residues 388–400 of SERT, *i.e.*, targeting the same region of EL4 recognized by 8B6 scFv (3). The antibody was tested in a protocol identical to that employed for the 15B8 Fab and 8B6 scFv. When cells were superfused with concentrations of the antiSERT-EL4 ranging from 1 to 30 nM prior to 5-HT application, we observed a concentration-dependent reduction in the peak current elicited by 5-HT (Fig. 3*A*). A plot of the peak currents, normalized to the amplitudes of the reference current, against the concentration of the antiSERT-EL4 antibody again resulted in a monophasic inhibition curve. Nonlinear regression gave an IC_50_ estimate of 2.2 ± 0.4 nM (mean ± SD) for the antiSERT-EL4 antibody (Fig. 3*B*). These data indicate that it is the occupation of EL4 that impedes the initial substrate-induced conformational changes in SERT.

### Binding kinetics of 8B6 scFv and antiSERT-EL4 antibody

**Figure 4:**
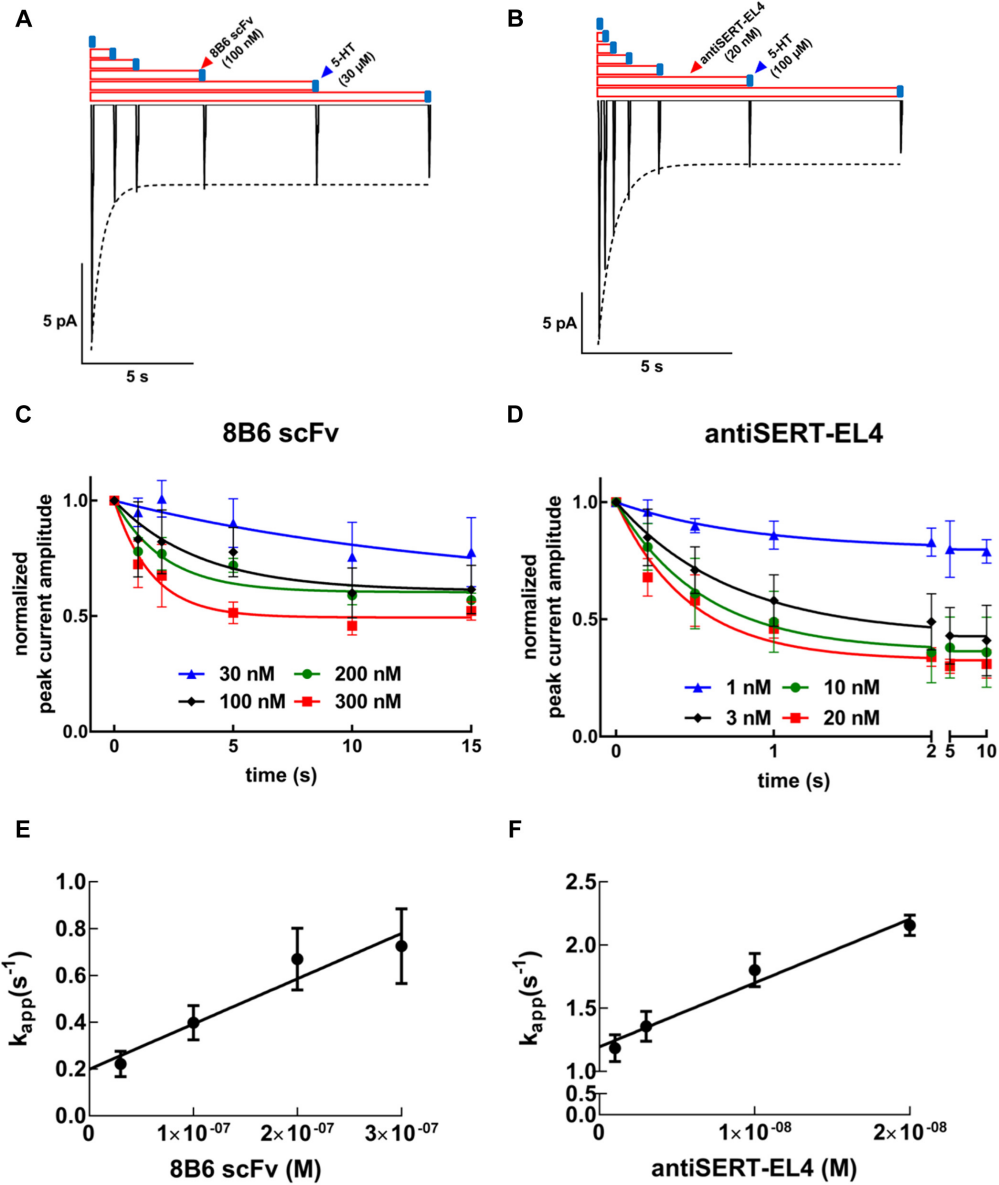
**Electrophysiological approach to measure the kinetics of 8B6 scFv- and antiSERT-EL4 antibody-binding to SERT.***A* and *B*, representative peak current traces recorded with variable intervals of preincubation (1, 2, 5, 10, and 15 s in *A* and 0.2, 0.5, 1, 2, 5, and 10 s in *B*) for 100 nM 8B6 scFv (*A*) and 20 nM antiSERT-EL4 antibody (*B*). *C* and *D*, normalized peak currents for the indicated concentrations of 8B6 scFv (*C*) and of antiSERT-EL4 antibody (*D*) were plotted as a function of time. The data points were fitted to the equation for a monoexponential decay to a basal level to estimate the apparent association rates (k_app_) for each concentration. These k_app_ values were plotted over the corresponding (*E*) 8B6 scFv and (*F*) antiSERT-EL4 antibody concentrations to yield a straight line. The data are mean ± SD from four to eight independent recordings. Association (k_on_) and dissociation (k_off_) rate constants were calculated from the slope and the *y*-intercept, respectively: k_on_-values for 8B6 scFv and antiSERT-EL4 were 1.93 ± 0.36 nM × 10^6^ M^−1^ s^−1^ and 5.05 ± 0.61 nM × 10^7^ M^−1^ s^−1^ and k_off_-values were 0.20 ± 0.07 s^−1^ and 1.20 ± 0.07 s^−1^, respectively.

We designed a time-course protocol to estimate the binding kinetics of 8B6 scFv and antiSERT-EL4 antibody. SERT-expressing cells attached to the recording pipet were exposed to 8B6 scFv (1, 2, 5, 10, and 15 s, cf*.* Fig. 4*A*) or antiSERT-EL4 antibody (0.2, 0.5, 1, 2, 5, and 10 s, cf*.* Fig. 4*B*). A time-dependent decrease in the peak current amplitude was observed for both the 8B6 scFv (Fig. 4*A*) and the antiSERT-EL4 antibody (Fig. 4*B*). For each concentration employed (*i.e.*, 30, 100, 200, and 300 nM 8B6 scFv or 1, 3, 10, and 20 nM antiSERT-EL4), the amplitudes of the peak currents were normalized to the preceding reference current and the normalized peak currents were plotted against exposure time. The resulting curves were fit by a monoexponential decay, which allowed for extracting the apparent association rate (k_app_) for each concentration of 8B6 scFv (Fig. 4*C*) and antiSERT-EL4 (Fig. 4*D*). As expected for a bimolecular reaction, the time-dependent reduction in peak current amplitude was accelerated with increasing 8B6 scFv or antiSERT-EL4 concentrations. The calculated values for k_app_ were (means ± SD) 0.22 ± 0.05 s^−1^, 0.40 ± 0.07 s^−1^, 0.67 ± 0.13 s^−1^, and 0.72 ± 0.12 s^−1^ for 30, 100, 200, and 300 nM 8B6 scFv, respectively, and 1.19 ± 0.30 s^−1^, 1.36 ± 0.26 s^−1^, 1.80 ± 0.32 s^−1^, and 2.16 ± 0.21 s^−1^ for 1, 3, 10, and 20 nM antiSERT-EL4 antibody, respectively.

A plot of k_app_ values for 8B6 scFv or antiSERT-EL4 *versus* concentration yielded straight lines, with the slopes and the *y*-intercepts of these straight lines corresponding to the association rate constants (k_on_) and the dissociation rates (k_off_), respectively. Thus, the k_on_ values for 8B6 scFv and antiSERT-EL4 (mean ± SD) were 1.93 ± 0.36 × 10^6^ M^−1^ s^−1^ and 5.05 ± 0.61 × 10^7^ M^−1^ s^−1^, and the k_off_ values were 0.20 ± 0.07 s^−1^ and 1.20 ± 0.07 s^−1^, respectively. Equilibrium dissociation constants (K_D_) for both the Fab and the antibody, calculated using the kinetic rate constants (k_off_/k_on_), were 103 nM and 23.8 nM, respectively. These kinetic K_D_ values for the 8B6 scFv and antiSERT-EL4 are not in line with their IC_50_ (25.3 and 4.2 nM) derived from the concentration–response curves summarized in Figures 2*B* and 3*B*. The actions of 8B6 scFv and antiSERT-EL4 antibody—*i.e.*, inhibition of the peak current in the absence of any appreciable effect on the steady-state current (Figs. 1, *A* and *B*, 2*A* and 3*A*)—are most readily accounted for by assuming that 8B6 scFv and antiSERT-EL4 antibody dissociate rapidly upon substrate binding to SERT. Thus, substrate binding to the 8B6 scFv or antiSERT-EL4 antibody-bound state of SERT promotes dissociation of the antibody (fragment) by forcing a conformation-dependent removal. Therefore, the calculated k_off_ and the K_D_ are a combination of antibody dissociation due to reversible binding and due to 5-HT-induced antibody release.

### Kinetic model for 8B6 scFv binding to SERT and 5-HT transport

**Figure 5:**
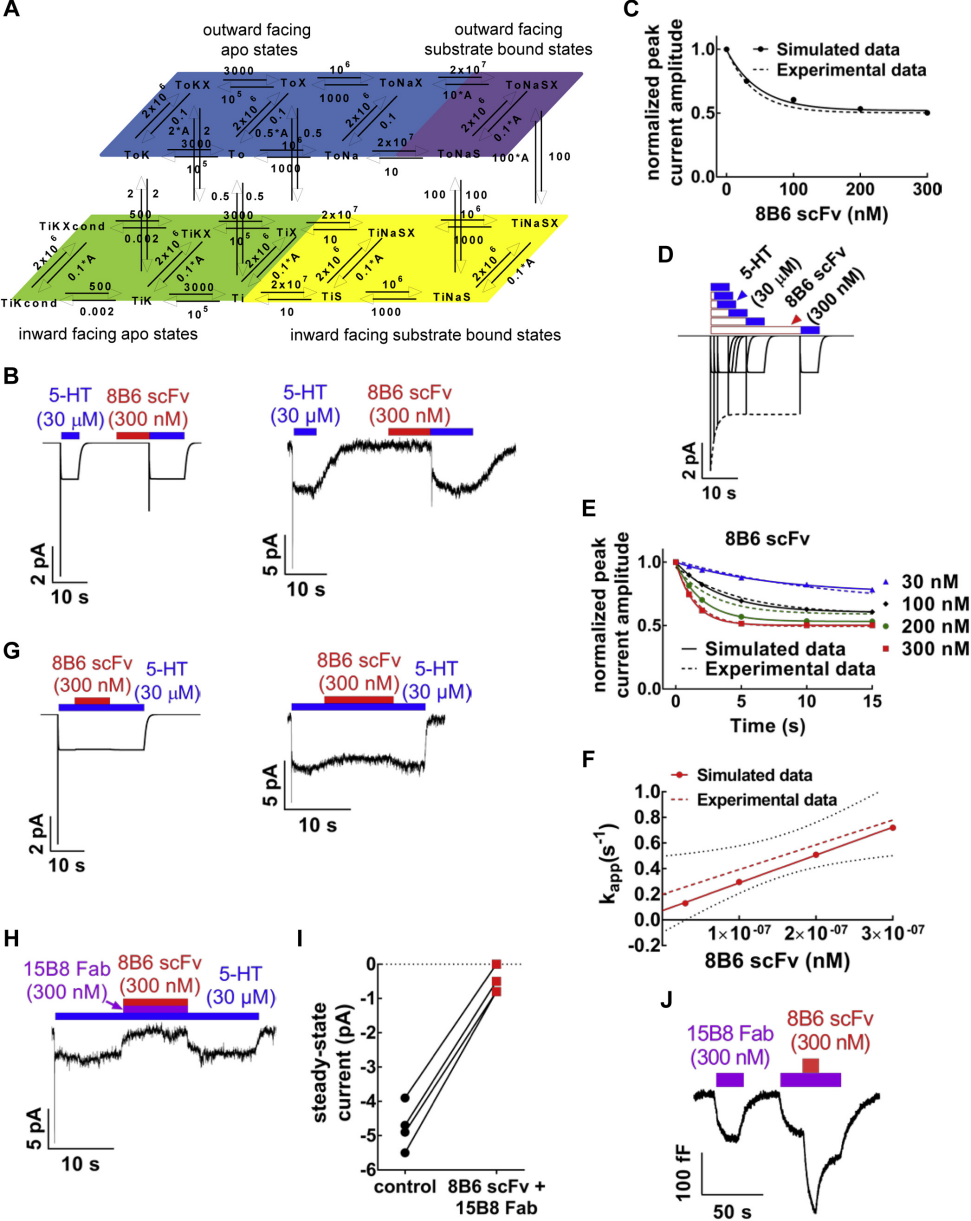
**Kinetic model for 8B6 scFv binding to SERT during the transport cycle.***A*, 8B6 scFv binding was explored during different conformational states of SERT (*blue:* outward-facing apo, *orange:* outward-facing substrate-bound, *green:* inward-facing apo, and *yellow:* inward-facing substrate-bound states). Kinetic rate constants for 8B6 scFv from Fig. 4 were implemented in the model. A is the factor describing the fold-acceleration of 8B6 scFv dissociation from SERT dwelling in the low-affinity states, where the outward-facing apo state is assumed to be the only high-affinity state for 8B6 scFv. X = 8B6 scFv, S = 5-HT. *B,* the synthetic current traces elicited by 30 μM 5-HT were generated by the model outlined in *panel A* using the ionic conditions employed in Figure 1, Figure 2, Figure 3 before and after 300 nM 8B6 scFv exposure. *C*, comparison of experimental (*dashed*) and synthetic (*solid*) data for the normalized reduction in the peak current amplitude by binding of 8B6 scFv to SERT. Simulations were done as shown in *panel B* for each indicated concentration 8B6 scFv. The experimental data are from Figure 2*B*. The IC_50_ calculated from the simulated data is 32.9 nM (95% confidence interval = 12.6–101.5 nM). *D*, compiled synthetic traces elicited by 30 μM 5-HT after 300 nM 8B6 scFv had been allowed to bind for 1, 2, 5, 10, or 15 s. The dashed line represents the monoexponential function fitted to the peak currents generated by the simulation. This curve was used to calculate k_app_. *E*, simulations were done as shown in *panel D* with the indicated concentrations of 8B6 scFv to extract the time-dependent decline in normalized peak current amplitudes. The monoexponential decay curves resulting from these simulations were plotted as *solid lines*. For comparison, the dashed lines show the curves generated by fitting the experimental data (taken from Fig. 4*C*). *F*, the k_app_ values extracted from the synthetic data shown in *Panel E* were plotted over the corresponding concentrations of 8B6 scFv to yield a straight line (*solid*). The *dashed line* indicates the linear regression to the experimental data (taken from Fig. 4*E*). The slope of the line through the synthetic points yields k_on_ = 1.93 × 10^6^ M^−1^∗s^−1^ and a *y*-intercept corresponding to k_off_ = 0.20 s^−1^. *G*, synthetic (*left panel*) and experimental traces (*right panel*) to highlight the modest inhibitory effect of 8B6 scFv on the 5-HT-induced steady-state current. The synthetic current traces elicited by 30 μM 5-HT and the effect of 300 nM 8B6 scFv wash-in and washout were generated by the model outlined in *panel A* using the ionic conditions employed in Figure 1, Figure 2, Figure 3. *H,* representative trace of the inhibition of the steady-state current by the simultaneous application of 8B6 scFv and 15B8 Fab using the recording conditions employed in Figure 1, Figure 2, Figure 3. *I,* Spaghetti plot from four independent recordings of steady-state current amplitudes done as in *panel H* before and after 8B6 scFv and 15B8 Fab application. *J,* representative trace of the apparent decrease in the membrane capacitance of HEK293 cells stably expressing GFP-tagged SERT upon consecutive binding of 15B8 Fab and 8B6 scFv. The recording condition was as in Fig. 1, *E* and *F*. The experiment was reproduced in two additional independent recordings.

We inferred that the discrepancy between IC_50_ and K_D_ values resulted from substrate-induced rapid dissociation of the 8B6 scFv and the EL4-directed antibody. We verified this assumption by developing a kinetic model for the transport cycle of SERT in the presence of the 8B6 scFv based on the kinetic rate constants calculated from the time-course experiments (Fig. 5*A*). The principles underlying this kinetic modeling have been described elsewhere (9). Our model posited that the 8B6 scFv bound in a conformation-dependent manner by favoring the outward-facing conformation of SERT in its apo-state: the dissociation of 8B6 scFv from SERT was predicted to be 100 times faster in the presence of 5-HT (factor "A" in Fig. 5*A*). This prediction was in line with the approximately fivefold difference between the IC_50_ and K_D_ in our experimental data, supporting our hypothesis of substrate-induced rapid dissociation of antibody. We carried out simulations using our model to confirm its validity based on our experimental data. In Figure 5*B*, the peak current protocol used in Figure 1, Figure 2, Figure 3 was applied to our modeled transporter *in silico* to examine the effect of 8B6 scFv on the peak current. The resulting synthetic traces (left panel in Fig. 5*B*) recapitulated the experimental data (right panel in Fig. 5*B*). We simulated experiments, which examined the reduction in peak current by 8B6 scFv over the same concentration range as in Figure 2*B*, *i.e.*, from 30 to 300 nM. The size of the peak currents was extracted from these *in silico* experiments, normalized to the peak current simulated in the absence of the antibody fragment, and plotted against the 8B6 scFv concentration (Fig. 5*C*). Our simulated modeling predicted an IC_50_ of 32.9 nM, thus the resulting inhibition curve (solid line) recapitulated the experimental data summarized in Figure 2*B* (its position is indicated by the dashed line in Fig. 5*C*).

We also interrogated the kinetic model to simulate the time-dependent inhibition of the peak current by increasing concentrations of 8B6 scFv. Figure 5*D* illustrates a representative synthetic experiment, which mirrored the experimental results found in Figure 4*A*: it is evident that the peak current declined with a monophasic exponential decay as the preincubation with 300 nM 8B6 scFv was increased. Similar synthetic experiments were done for 30, 100, and 200 nM 8B6 scFv, and the time course of peak current inhibition was compared with that observed in the actual experiments: the *in silico* data (Fig. 5*E*) recapitulated the experimental findings with reasonable fidelity (taken from Fig. 4*C* and shown as dashed lines in Fig. 5*E*). A curve of monoexponential decay was fitted to the synthetic data for each 8B6 scFv concentration to extract the apparent on-rate k_app_ (Fig. 5*E*, solid lines). The calculated k_app_ values of the *in silico* data were 0.08 s^−1^, 0.21 s^−1^, 0.35 s^−1^, and 0.62 s^−1^ for 30, 100, 200, and 300 nM 8B6 scFv, respectively. A plot of the k_app_ values of the *in silico* data over the corresponding 8B6 scFv concentrations yielded a straight line (solid line in Fig. 5*F*). For comparison, the linear regression to the experimental data (taken from Fig. 4*E*) and the 95% confidence interval are also shown in Figure 5*F* as dashed lines. We derived k_on_ (2.17 × 10^6^ M^−1^ s^−1^) and k_off_ (0.07 s^−1^) for 8B6 scFv from the simulated data. The bimolecular association rate constant k_on_ (1.93 × 10^6^ M^−1^ s^−1^) estimated from the actual experiments is in line with the k_on_ determined from the simulation. In contrast, there is a discrepancy between the k_off_-values estimated from the experimental data (0.20 s^−1^) and the k_off_ extracted from the simulation, which is due to the low precision in estimating the *y*-intercept (cf*.* 95% confidence interval in Fig. 5*F*).

Finally, we examined the ability of 8B6 scFv to bind to SERT under steady state-conditions during the transport cycle (left panel in Fig. 5*G*): a hypothetical cell expressing SERT was superfused with 5-HT for 25 s. Following an interval of 5 s, after we recorded the reference peak and the steady-state components, 8B6 scFv was coapplied with the substrate for 10 s to observe its effect on the steady-state current. Then, 8B6 scFv was washed out by buffer containing only the substrate for 10 s, allowing for return to the reference steady-state component. At last, the substrate was also removed by washing with buffer until the signal reached the baseline. The model generated a synthetic current trace, where 8B6 scFv (300 nM) only caused a very small inhibition of the steady-state current. This is consistent with the fact that the binding of 8B6 scFv to SERT is not favored in the presence of the substrate. More importantly, we also carried out actual experiments to verify the prediction of the model. It is evident from the representative trace shown in the right panel of Figure 5*G* that the experimental observations matched the *in silico* data closely: we observed only a very modest inhibition of the steady-state current by 8B6 scFv (300 nM). The combined application of 8B6 scFv and 15B8 Fab (each at 1 μM) was previously shown to fully block substrate uptake by SERT (3). We verified that this inhibition was recapitulated in electrophysiological recordings: the representative trace in Figure 5*H* shows that in a cell, which was superfused with 15B8 Fab, addition of 8B6 scFv resulted in a pronounced suppression of the steady-state current. This inhibition was promptly reversed by washout of 8B6 scFv. On average the combination of 15B8 Fab and 8B6 scFv (each at 300 nM) suppressed the current by about 90% (Fig. 5*I*). We independently confirmed by capacitance recordings that both antibodies were bound simultaneously (Fig. 5*J*).

### Saturation binding of radiolabeled inhibitors to SERT in the presence of EL4-and EL2-directed antibody (fragments)

**Figure 6:**
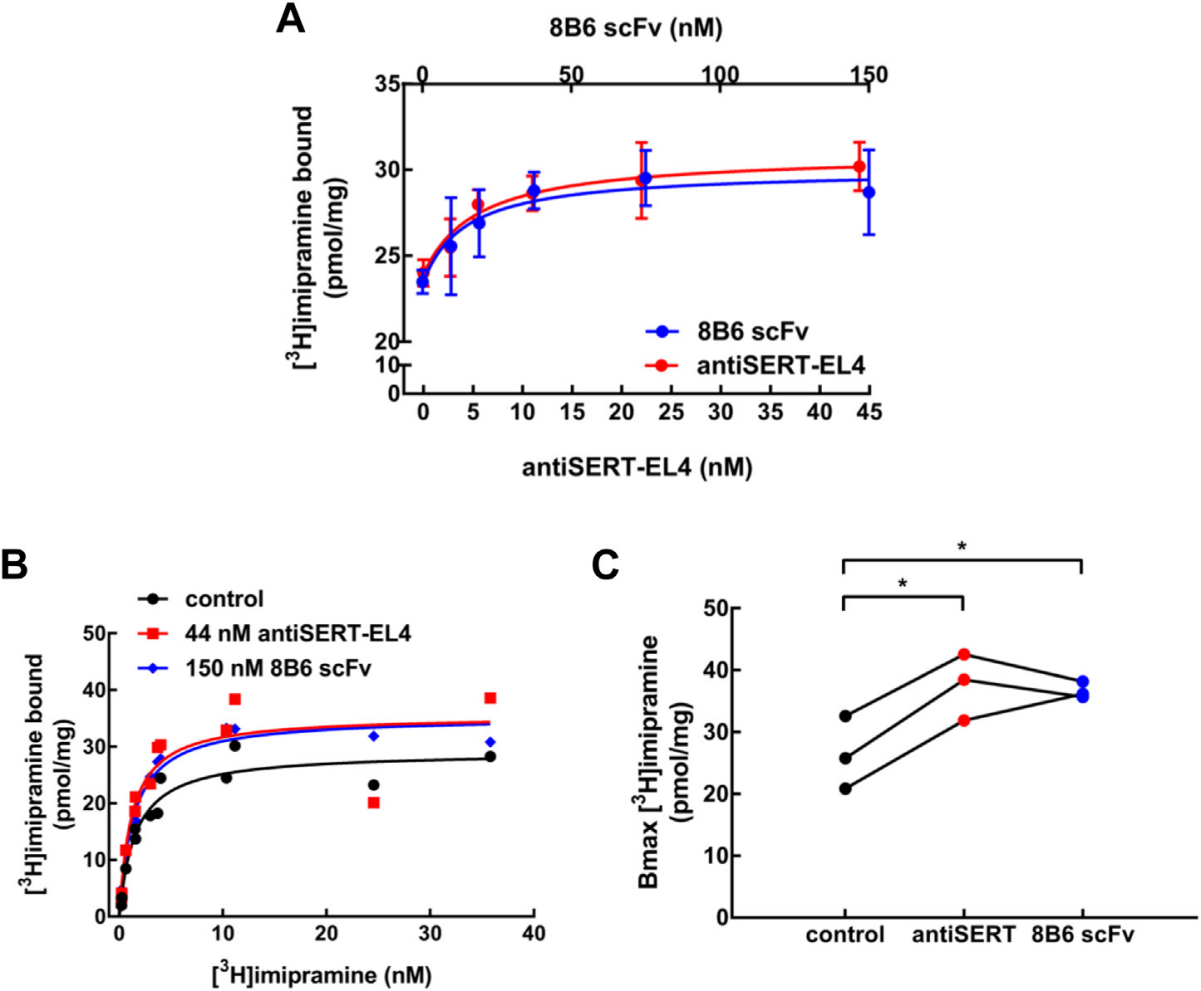
**Binding of [**^**3**^**H]imipramine to SERT in the absence and presence of 8B6 scFv or of the antiSERT-EL4 antibody.***A*, concentration–response curve for 8B6 scFv and antiSERT-EL4 antibody in enhancing binding of [^3^H]imipramine to SERT. The reaction was done in a final volume of 0.2 ml containing membranes from HEK293 cells stably expressing GFP-tagged SERT (2 μg). 8B6 scFv (9.5, 19, 37.5, 75, and 150 nM, denoted on the top *x*-axis) and antiSERT-EL4 (2.7, 5.5, 11, 22, and 44 nM, denoted on the bottom *x*-axis) were preincubated with the membranes for 30 min at 25 °C. The binding reaction was carried out in the presence of 10 nM [^3^H]imipramine for 10 min at 25 °C, as outlined under Materials and Methods. Data are means ± SD from three independent experiments carried out in duplicate. The lines were drawn by fitting the data to the equation for a rectangular hyperbola + basal binding in the absence of Fab/antibody. EC_50_ values for 8B6 scFv and antiSERT-EL4 antibody were 14.9 ± 2.0 nM and 4.9 ± 0.6 nM, respectively. *B*, saturation of [^3^H]imipramine binding to SERT in the absence and presence of 8B6 scFv (150 nM) or antiSERT-EL4 (44 nM). The reaction was done as in *panel A* with [^3^H]imipramine concentrations ranging from 0.2 to 36 nM. Shown is a representative experiment performed in duplicate. The lines were drawn by fitting the data to a rectangular hyperbola. *C*, the spaghetti plot depicts the change in B_max_ of [^3^H]imipramine binding in the presence of 8B6 scFv or antiSERT-EL4 antibody in three independent experiments carried out in duplicate (statistical significance was tested by repeated-measures ANOVA, followed by Holm–Sidak's multiple comparisons; B_max_ in the presence of 8B6 scFv or of antiSERT-EL4 antibody differed from control B_max_, *p* < 0.05).

Taken together, the experimental and *in silico* kinetics show that 8B6 scFv and the antiSERT-EL4 antibodies bind to the outward-facing conformation of SERT. EL4 of LeuT, the bacterial ortholog of SERT, is proposed to act as a “lid” on the binding site (19). The X-ray crystal structure indicates that EL4 is located in a similar position in SERT (3). Thus, we surmised that the movement of EL4 was restricted when 8B6 scFv or antiSERT-EL4 was bound, rendering the binding site of SERT more accessible to ligands. We tested this hypothesis in binding experiments with the inhibitor [^3^H]imipramine. The binding of [^3^H]imipramine to the membranes harboring GFP-tagged SERT was enhanced by 8B6 scFv and by antiSERT-EL4 antibody in a concentration-dependent manner (Fig. 6*A*). The EC_50_ values for 8B6 scFv and by antiSERT-EL4 antibody were 14.9 ± 2.0 nM and 4.9 ± 0.6 nM, respectively. These EC_50_-values are reasonably similar to the IC_50_ estimates obtained by electrophysiological recordings (cf*.* Figs. 2*B* and 3*B*), but differ from the kinetic K_D_ values (cf*.* Fig. 4, *E* and *F*). Surprisingly, in saturation experiments, the effect of 8B6 scFv (150 nM) or antiSERT-EL4 (44 nM) was accounted for by an increase in B_max_ (Fig. 6, *B* and *C*) rather than by a change in dissociation constant: the K_D_-values in the presence of 8B6 scFv (1.20 ± 0.43 nM) and of the antibody (1.38 ± 0.17 nM) were comparable to that seen in their absence (control K_D_ = 1.55 ± 0.22 nM).

**Figure 7:**
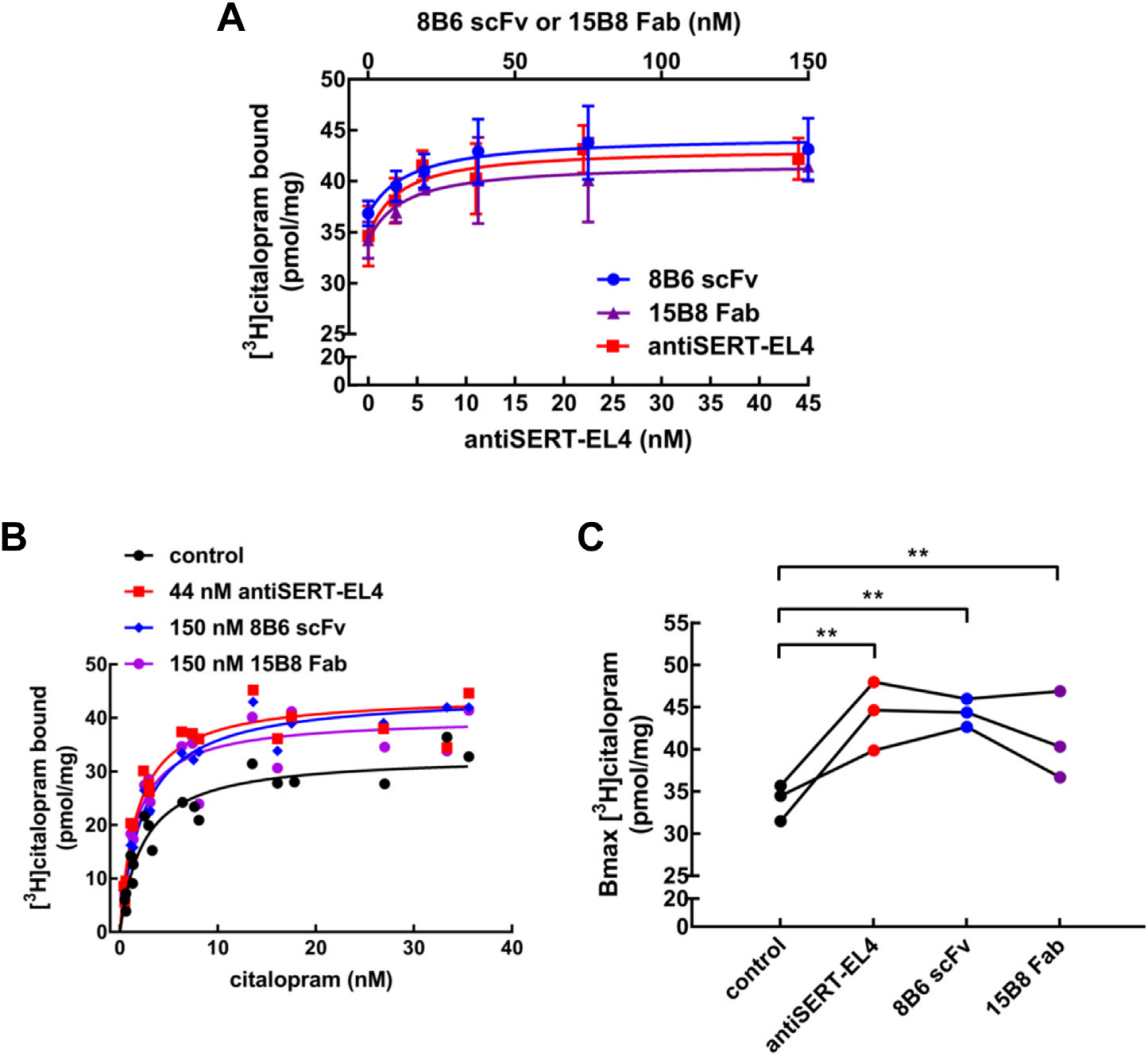
**Binding of [**^**3**^**H]citalopram to SERT in the presence of 8B6 scFv, 15B8 Fab, or of the antiSERT-EL4 antibody.***A*, concentration–response curve for 8B6 scFv, 15B8 Fab, and antiSERT-EL4 antibody in enhancing binding of [^3^H]citalopram to SERT. The experiments were carried out as outlined for [^3^H]imipramine binding in Figure 6*C*. The concentration of [^3^H]citalopram was 10 nM. The concentrations were 9.5, 19, 37.5, 75, and 150 nM for both 8B6 scFv and 15B8 Fab (denoted on the top *x*-axis), whereas the antiSERT-EL4 antibody concentrations were 2.7, 5.5, 11, 22, and 44 nM (denoted on the bottom *x*-axis). Data are means ± SD from three independent experiments carried out in duplicate. The lines were drawn by fitting the data to the equation for a rectangular hyperbola + basal binding in the absence of Fab/antibody. EC_50_ values were 14.7 ± 2.3 nM, 11.2 ± 2.5 nM, and 5.0 ± 2.7 nM for 8B6 scFv, 15B8 Fab, and antiSERT-EL4, respectively. *B*, saturation of [^3^H]citalopram binding to SERT in the absence and presence of 8B6 scFv (150 nM), 15B8 Fab (150 nM), or antiSERT-EL4 (44 nM). The reaction was done as in *panel A* with [^3^H]citalopram concentrations ranging from 0.4 to 36 nM. Shown are the pooled data from three experiments carried out in duplicate. The lines were drawn by fitting the data to a rectangular hyperbola. *C*, the spaghetti plot depicts the change in B_max_ of [^3^H]citalopram binding in the presence of 8B6 scFv, 15B8 Fab, or antiSERT-EL4 antibody in three independent experiments carried out in duplicate (statistical significance was tested by repeated-measures ANOVA, followed by Holm–Sidak's multiple comparisons; B_max_ in the presence of 8B6 scFv, 15B8 Fab, or of antiSERT-EL4 antibody differed from control B_max_, *p* < 0.005).

The increase in binding was confirmed by determining the effects of 8B6 scFv and of the antiSERT-EL4 antibody on [^3^H]citalopram, another radiolabeled SERT inhibitor. Addition of either 8B6 scFv or the antiSERT-EL4 antibody resulted in concentration-dependent enhancement binding of [^3^H]citalopram to SERT (Fig. 7*A*). We also interrogated the role of EL2 by examining the effect of 15B8 Fab. As shown in Figure 7*A*, the 15B8 Fab also promoted binding of [^3^H]citalopram to SERT, the EC_50_-values for 8B6 scFv, 15B8 Fab, and the antiSERT-EL4 antibody were 14.7 ± 2.3 nM, 11.2 ± 2.5 nM and 5.0 ± 2.7 nM for 8B6 scFv, 15B8 Fab, and antiSERT-EL4, respectively. Again, the EC_50_-values for 8B6 scFv and the antiSERT-EL4 antibody were in line with the EC_50_-values calculated from imipramine-binding experiments and IC_50_-values from whole-cell patch-clamp recordings. In saturation experiments, the increase in B_max_, which was observed for imipramine binding (cf. Fig. 6, *B* and *C*), was recapitulated in the presence of 8B6 scFv and the antiSERT-EL4 antibody, and it was also observed with 15B8 Fab (Fig. 7, *B* and *C*). We again failed to detect any change in affinity for [^3^H]citalopram binding in the presence of 8B6 scFv (K_D_ = 2.40 ± 0.24 nM) when compared with the control in the absence of antibody (K_D_ = 2.36 ± 0.23 nM). However, a modest increase in affinity of [^3^H]citalopram for SERT was detectable in the presence of the antiSERT-EL4 antibody (K_D_ = 1.73 ± 0.22 nM) and of 15B8 Fab (K_D_ = 1.60 ± 0.23 nM).

### Binding kinetics of [^3^H]citalopram to SERT in the presence of 8B6 scFv, 15B8 Fab, and antiSERT-EL4 antibody

**Figure 8:**
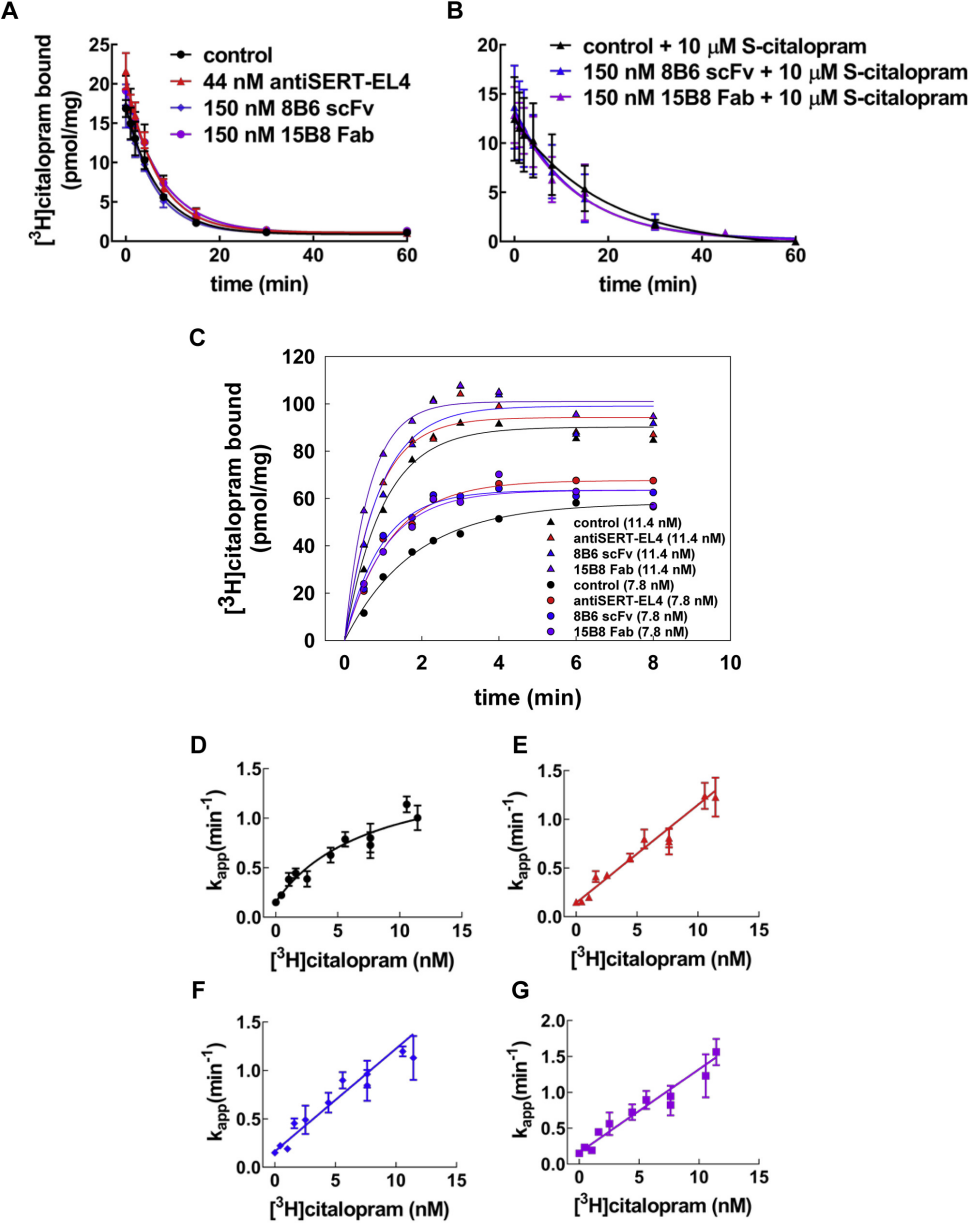
**Kinetics of [**^**3**^**H]citalopram binding to SERT in the presence of 8B6 scFv, 15B8 Fab, or antiSERT-EL4 antibody.***A*, dissociation experiment: membranes harboring GFP-tagged SERT (1 μg/assay) were incubated with [^3^H]citalopram (6 nM) in the presence of 8B6 scFv (150 nM), 15B8 Fab (150 nM), or antiSERT-EL4a antibody (44 nM) in a final volume of 10 μl. After 10 min at 25 °C, the dissociation was initiated by 100-fold dilution of the reaction in buffer containing the same concentration of 8B6 scFv, 15B8 Fab, or antiSERT-EL4 antibody. Data are means ± SD from three independent experiments carried out in duplicate. The lines were drawn by fitting the data to the equation for a monoexponential decay. *B*, dissociation of [^3^H]citalopram in the presence of 10 μM S-citalopram. The experiment was done as in *panel A* with 5 nM [^3^H]citalopram, but 10 μM S-citalopram was present in in the dilution buffer to occupy the S1 site and to thereby slow dissociation of prebound [^3^H]citalopram. The parallel incubations were done in the absence of S-citalopram. These are not shown for the sake of clarity, but the dissociation rate constants were identical to those determined in *panel A*. Data are means ± SD from three independent experiments carried out in duplicate. *C*, association experiment: membranes harboring GFP-tagged SERT (1 μg/assay) were preincubated with 8B6 scFv (150 nM), 15B8 Fab (150 nM), or antiSERT-EL4 antibody (44 nM) for 10 min at 25 °C. Thereafter the binding reaction (final volume 0.1 ml) was initiated by the addition of [^3^H]citalopram (7.8 nM and 11.4 nM) and terminated by rapid filtration at the indicated time points, bound to membranes prior to the binding reaction. The lines were drawn by fitting the data to the equation for a monoexponential association. Data are means from duplicate determinations in a representative experiment, which was carried out in parallel. *D–F*, the apparent association rates (k_app_ ± SE) were obtained from experiments done as shown in *panel C* and plotted as a function of the [^3^H]citalopram concentration plots. Under control conditions (*D*), *i.e.*, in the absence of antibody/Fabs, the resulting plot was better described by the sum of a rectangular hyperbola and a basal term (= k_off_) than by a straight line (*p* = 0.02; F-test based on the extra-sum-of-squares principle). In contrast, in the presence of antiSERT-EL4 antibody (*E*), 8B6 scFv (*F*), and 15B8 Fab (*G*), the linear regression (with k_off_ as *y*-intercept and k_on_ as slope) was an adequate and parsimonious description because there was not any improvement in the fit by assuming a hyperbolic relation.

Saturation experiments may not be sensitive enough to detect modest changes in K_D_. Accordingly, we measured the kinetics of inhibitor binding to SERT in the presence of 8B6 scFv, 15B8 Fab, or antiSERT-EL4 antibody. We selected [^3^H]citalopram as the radioligand because it has a much slower dissociation rate constant than [^3^H]imipramine (23). Thus, k_off_ can be determined with adequate precision, and the signal-to-noise ratio is large to detect both an increase and a decrease in k_off_. The binding of [^3^H]citalopram to SERT was allowed to reach equilibrium in the presence of 8B6 scFv, 15B8 Fab, or antiSERT-EL4 antibody, and dissociation was subsequently initiated by a 100-fold dilution of the radioligand, without dilution of the antibody/Fabs: k_off_ values in the presence of 8B6 scFv (0.16 ± 0.02 min^−1^; mean ± SD), 15B8 Fab (0.13 ± 0.02 min^−1^), or antiSERT-EL4 (0.15 ± 0.02 min^−1^) were comparable to those under control conditions (0.15± 0.02 min^−1^) (Fig. 8*A*). Occupancy of the vestibular S2 site by micromolar concentrations of citalopram delays dissociation of [^3^H]citalopram from the S1 site (16, 23). It is conceivable that binding of antibodies to EL2 and EL4 of SERT exerts its action *via* enhancing binding to the S2 site. Accordingly, we determined the dissociation of prebound [^3^H]citalopram after dilution into buffer containing 10 μM S-citalopram. This concentration is within the steep part of the concentration–response curve (16). Thus it allows for detecting an additional decline in the dissociation rate in the presence of the antibodies (if they enhance the affinity to the S2 site) or an inhibition of the allosteric effect (if the antibodies limit access to S2 site). Under control conditions, 10 μM S-citalopram reduced the dissociation rate of prebound [^3^H]citalopram (k_off_ to 0.06 ± 0.01 min^−1^, black triangles in Fig. 5*B*), *i.e.*, to an extent comparable to that previously reported (16). The allosteric action of S-citalopram was also seen in the presence of 8B6 scFv or of 15B8 Fab with k_off_ values of 0.07 ± 0.01 min^−1^ and 0.07 ± 0.01 min^−1^, respectively (Fig. 5*B*).

In contrast, the rate of the forward-binding reaction was accelerated, if membranes harboring GFP-tagged SERT were preincubated with saturating concentrations of 8B6 scFv, 15B8 Fab, or antiSERT-EL4 antibody and the binding reaction subsequently initiated by the addition of [^3^H]citalopram to yield 7.8 or 11.4 nM (Fig. 8*B*). We carried out experiments with [^3^H]citalopram concentrations covering the range of 0.45–11 nM to explore the relation between radioligand concentration and the apparent association rate (k_app_). For a simple bimolecular reaction, k_app_ depends linearly on ligand concentration. However, there was a hyperbolic relation under control conditions, *i.e.*, in the absence of the antibody or the Fabs: the rate of binding at high [^3^H]citalopram was slower than extrapolated from the pseudo-first-order rate measured at low concentration (Fig. 8*C*). In contrast, over the concentration range tested, k_app_ depended in a linear manner on the [^3^H]citalopram concentration in the presence of the antiSERT-EL4 antibody (Fig. 8*D*), 8B6 scFv (Fig. 8*E*), or 15B8 Fab (Fig. 8*F*).

### Occupancy by 15B8 Fab of EL2 accelerates binding of methylphenidate to SERT

**Figure 9:**
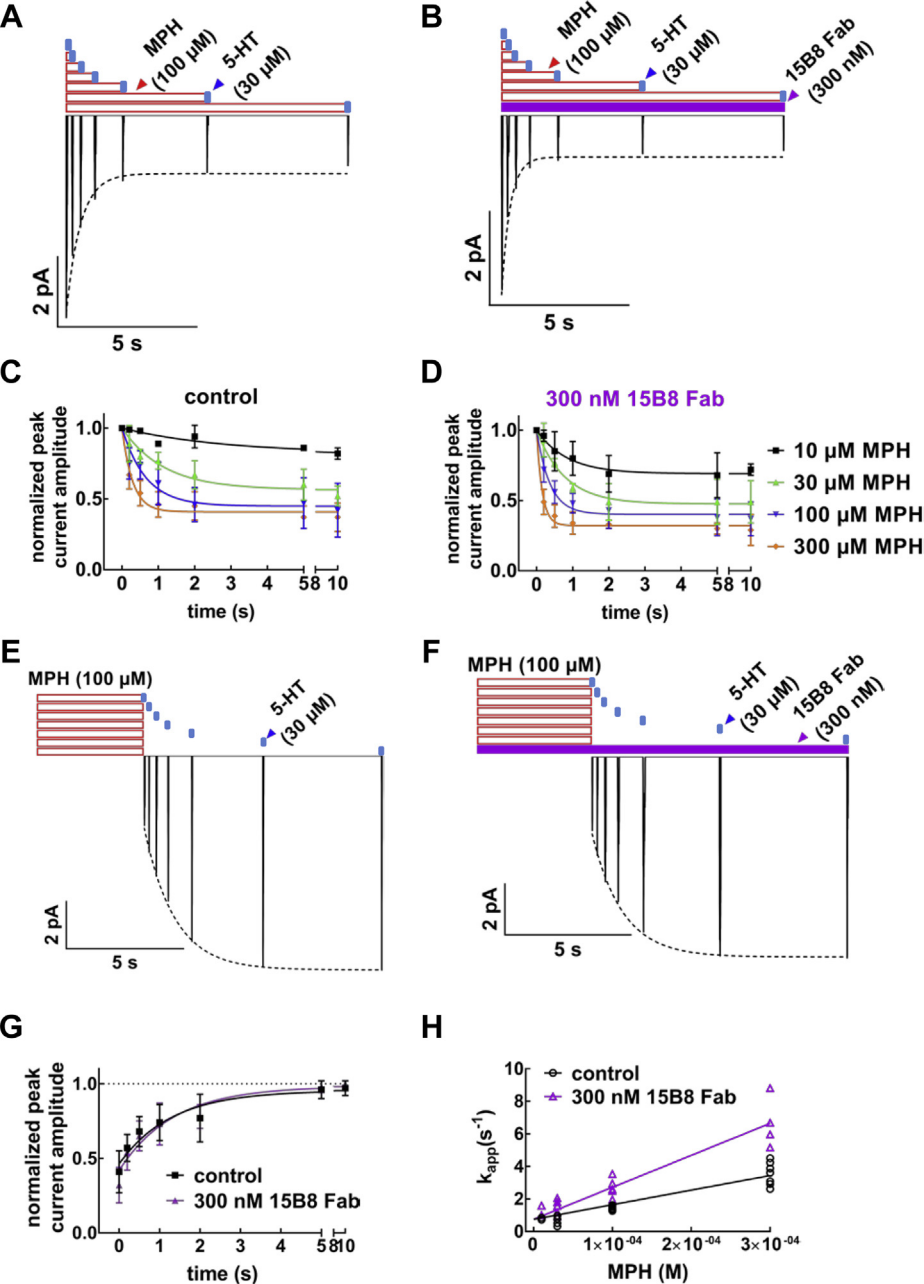
**Electrophysiological approach to measure the kinetics of methylphenidate (MPH) binding to SERT in the absence and presence of the 15B8 Fab.***A* and *B,* time course for peak current inhibition by MPH in the absence (*A*) and presence of 15B8 Fab (*B*). HEK293 cells stably expressing GFP-tagged SERT were superfused with 30 μM 5-HT to elicit the reference peak current and subsequently superfused with 100 μM methylphenidate (MPH) for 0.2, 0.5, 1, 2, 5, and 10 s in the absence (*A*) or presence of 300 nM 15B8 Fab (*B*) prior to eliciting the test peak current with 30 μM 5-HT. Peak currents were isolated (*i.e.*, the steady-state current was suppressed) by a high concentration of Na^+^ (152 mM) in the internal solution in the patch pipet. Shown is the compilation of representative peak current traces from a single cell. The time course of current decay reflects the apparent association rate of methylphenidate binding and blockage of SERT. The *dashed lines* were drawn by fitting the decline in peak current amplitude to an equation for a monoexponential decay to a residual activity. *C* and *D*, the experiments were carried out as outlined in *panels A* and *B* with 10, 30, 100, and 300 μM methylphenidate (MPH) in the absence (*C*) and presence of 300 nM 15B8 Fab 8 (*D*). The peak current reduction was normalized by dividing test currents by the reference current and plotted as a function of time. Data are means ± SD from three to seven independent recordings for each concentration of methylphenidate. *E* and *F*, time course of peak current recovery after methylphenidate washout. HEK293 cells stably expressing GFP-tagged SERT were superfused with 100 μM methylphenidate (MPH) for 5 s in the absence (*E*) or presence (*F*) of 300 nM 15B8 Fab. Then, the peak currents were elicited by rapid application of 30 μM 5-HT with 0, 0.2, 0.5, 1, 2, 5, and 10 s delay to observe its recovery. The dashed lines denote the monoexponential fit for peak current recovery resulting from the dissociation of methylphenidate. The k_off_ of methylphenidate was extracted from this fit. *G*, the time course of the peak current recovery after washout of methylphenidate (MPH) was determined as depicted in Figure 9, *E* and *F* in the absence of SERT (*black*) and presence of 15B8 Fab-bound (*purple*). The solid curves were generated by fitting the data to the equation for a monoexponential rise from a basal residual peak current to obtain k_off_. The data are means ± SD from 13 independent recordings for both conditions. *H*, the values for k_app_ were extracted from *panels C* and *D* and plotted as a function of methylphenidate (MPH) concentrations. Dissociation rates (k_off_) were taken from *panel G* to constrain the *y*-intercepts of the lines in the linear regression. The k_on_ of methylphenidate from the slope of the lines in the absence (*black:* control) and presence of 15B8 Fab (300 nM, *purple*). The rates for methylphenidate association (k_on_) in the absence and presence of 15B8 Fab were 8.95 ± 1.03 × 10^3^ M^−1^ s^−1^ and 1.96 ± 0.06 × 10^4^ M^−1^ s^−1^, respectively.

The data summarized in Figure 6, Figure 7, Figure 8 indicate that restricting the conformational flexibility of EL2 and EL4 by binding of antibody enhances the accessibility of the binding site for inhibitors. Methylphenidate is a selective inhibitor of the dopamine transporter (DAT/SLC6A3). The low affinity of methylphenidate for SERT is determined by a slow association rate. Once bound to the binding pocket, methylphenidate is released with equivalent dissociation rates from DAT and SERT (18). Accordingly, we explored the hypothesis that restricting the flexibility of extracellular loops may increase the affinity of methylphenidate. In binding experiments with [^3^H]imipramine, we observed a shift to the left of the methylphenidate competition curve in the presence of 8B6 scFv, 15B8 Fab, or antiSERT-EL4 antibody (data not shown). However, because of uncertainty in the affinity of the radioligand in the presence of antibody and Fabs (cf. Fig. 6), this apparent shift in affinity is difficult to interpret. In addition, it does not provide kinetic information. We therefore resorted to an electrophysiological approach, which relies on the substrate-induced peak current through SERT for determining the occupancy of the binding site by methylphenidate. 8B6 scFv and antiSERT-EL4 antibody reduced the peak current (cf. Figure 1, Figure 2, Figure 3, Figure 4, Figure 5), but 15B8 Fab did not (cf*.* Fig. 1). 15B8 Fab, however, enhanced ligand binding in a manner similar to 8B6 scFv and the antiSERT-EL4 antibody. Hence, we examined the actions of 15B8 Fab on the onset of peak current inhibition by methylphenidate (Fig. 9, *A*–*D*) and on recovery from peak current inhibition by methylphenidate (Fig. 9, *E*–*G*). The recordings were done in the presence of high internal Na^+^ (152 mM) because a high internal Na^+^ concentration eliminates the steady-state current component (5). The isolated peak current can be quantified with high precision (5). The association rate of methylphenidate in the absence (Fig. 9*A*) and presence (Fig. 9*B*) of 15B8 Fab was measured by applying 5-HT (30 μM) for 3 s to obtain the reference current. Cells were subsequently exposed to methylphenidate for time intervals varying between 0.2, 0.5, 1, 2, 5, and 10 s prior to eliciting a current by fresh superfusion with 5-HT. This allowed for monitoring the onset of the methylphenidate-induced inhibition of the peak current. When plotting the recorded peak currents normalized to the reference current against exposure time, we estimated apparent on-rates (k_app_) for 10, 30, 100, and 300 μM methylphenidate in the range of 0.36 ± 0.12 s^−1^, 0.87 ± 0.20 s^−1^, 1.53 ± 0.40 s^−1^, and 3.46 ± 0.51 s^−1^ in the absence (Fig. 9*C*) and 1.20 ± 0.42 s^−1^, 1.29 ± 0.27 s^−1^, 2.72 ± 0.45 s^−1^, and 6.54 ± 1.04 s^−1^ in the presence of 300 nM 15B8 Fab (Fig. 9*D*), respectively (mean ± SD).

The off-rate of methylphenidate was measured by reversing the order, *i.e.*, cells were first superfused for 5 s with methylphenidate in the absence (Fig. 9*E*) and in the presence of 300 nM 15B8 Fab (Fig. 9*F*) followed by a washout with buffer lacking or containing 15B8 Fab for 0.2, 0.5, 1, 2, 5, and 10 s. As expected, as a result of methylphenidate dissociation induced by its washout, the peak currents recovered in a time-dependent manner. In contrast to the onset of peak current inhibition (Fig. 9, *A*–*D*), the presence of 15B8 Fab did not affect the time-dependent recovery of the peak current, which was adequately described by a monoexponential function (Fig. 9, *E*–*G*). Thus the estimated dissociation rates were comparable in the absence (k_off_ = 0.89 ± 0.14 s^−1^) and presence of 15B8 Fab (k_off_ = 0.80 ± 0.12 s^−1^).

When plotted as a function of methylphenidate concentration, k_app_-values increased in a linear manner (Fig. 9*H*); the *y*-intercept was consistent with the k_off_ extracted from the time-dependent peak current recovery. We calculated the association rate constant by linear regression with the *y*-intercept constrained to the measured k_off_: in the presence of 15B8 Fab, k_on_ for methylphenidate binding to SERT increased from 8.55 ± 1.03 × 10^3^ M^−1^ s^−1^ to 1.92 ± 0.06 × 10^4^ M^−1^ s^−1^. Therefore, restricting the conformational flexibility of EL2 by binding of 15B8 Fab translated into improved access of methylphenidate to the binding site of SERT.

## Discussion

Binding of most ligands to SERT (18, 24) and other monoamine transporters (18) proceeds at rates that are substantially below the rates imposed by diffusion. This typically indicates that the binding site is not readily accessible. Here we addressed the roles of the two large extracellular loops, *i.e.*, EL2 and EL4, in the conformational rearrangements associated with binding of substrates and inhibitors to SERT. Our analysis relied on an electrophysiological approach, which allowed for recording conformational transitions and binding events in real time. The binding of substrate and cosubstrate ions triggers a conformational change, which initiates their translocation across the membrane. This can be monitored by the resulting charge movement across the membrane electric field, which produces a transient peak current (5). Our findings support the following conclusions: (i) EL4 must move to support the conformational transition required for substrate translocation. This conclusion is based on the observation that both the antiSERT-EL4 antibody and 8B6 scFv reduced the peak current. In contrast, occupancy of EL2 by 15B8 Fab does not impede the initial conformational transitions of the transport cycle. (ii) The antiSERT-EL4 antibody and 8B6 scFv bind preferentially to EL4 to the outward-facing substrate-free (apo) state. This conclusion is also supported by the kinetic model, which provided synthetic traces that recapitulated all experimental data. Importantly, the kinetic model also predicted a very modest inhibition of the steady-state current, a prediction verified by actual recordings. Thus because of their low affinity to EL4 in the substrate-bound state, 8B6 scFv and the antiSERT-EL4 antibody cannot disrupt the transport cycle. These findings highlight the fact that during the transport cycle, EL4 must undergo a conformational rearrangement of a magnitude sufficient to promote release of the bound antibody and to preclude its binding. (iii) While initial substrate binding is only affected by antibody-induced restriction of EL4 movement, both EL2 and EL4 impinge on binding of typical inhibitors. This conclusion is based on two independent lines of evidence: all three antibody fragments/antibodies enhanced the binding of radiolabeled [^3^H]citalopram by enhancing its association rather than its dissociation rate. Electrophysiological recordings allowed for directly assessing the kinetics of methylphenidate binding; 15B8 Fab, the antibody fragment directed against EL2, accelerated the association but not the dissociation of methylphenidate. The selectivity of methylphenidate for DAT over SERT is determined by its association rates (18). Taken together, the observations show that EL2 and EL4 contribute to a selectivity filter in SERT.

There are two interpretations of the binding reaction of a ligand to a protein: in the induced fit model, the initial binding of the ligand induces a conformational change, which results in high-affinity binding (25). In the model of conformational selection, the protein isomerizes spontaneously and the ligand binds preferentially to one of the states, which are visited by the protein (26). All typical inhibitors bind to the outward-facing state of monoamine transporters (27). Thus, by definition, there is conformational selection during the binding reaction. In addition, SERT contains two binding sites, a vestibular S2 site and the primary binding site S1 (3). The two sites are allosterically linked (15, 28, 29). Vestibular low-affinity binding must precede high-affinity binding to S1. Hence, the slow association rate constant may also reflect an induced fit, which supports high-affinity binding to S1. In fact, we observed that in the absence of EL2-or EL4-directed antibodies, the apparent association rate k_app_ of [^3^H]citalopram did not increase in a linear manner when the concentration of radioligand was increased. Instead, there was a hyperbolic relation between k_app_ and [^3^H]citalopram concentration. While this hyperbolic relation is indicative of an induced fit model (30), it does not necessarily contradict conformational selection: it has been argued that, in many instances, the apparent induced fit can be treated as a special case of conformational selection (31). It is conceivable that the antibodies may affect the equilibrium between a state, which allows for high-affinity binding of the radioligand, and a state where binding is of low affinity and thus inaccessible to detection because of rapid dissociation. This scenario of conformational selection can account for the increase in B_max_, which was seen in the presence of antibodies. Thus, our data do not allow us to discriminate between induced fit and conformational selection. However, they unequivocally show that EL2- and EL4-directed antibodies relieve a constraint and facilitate binding of inhibitors. Thus EL2 and EL4 can be conceptualized as part of a selectivity filter in the extracellular gate, which limits access of inhibitors. Site-directed mutagenesis studies also point to a role of EL4 in determining the affinity for at least some inhibitors: substitution of L406 in EL4b by glutamate accelerates the association rate constant for [^3^H]citalopram but not [^125^I]RTI-55 (3β-(4-iodophenyl)tropan-2 beta-carboxylic acid methyl ester) (20).

**Figure 10:**
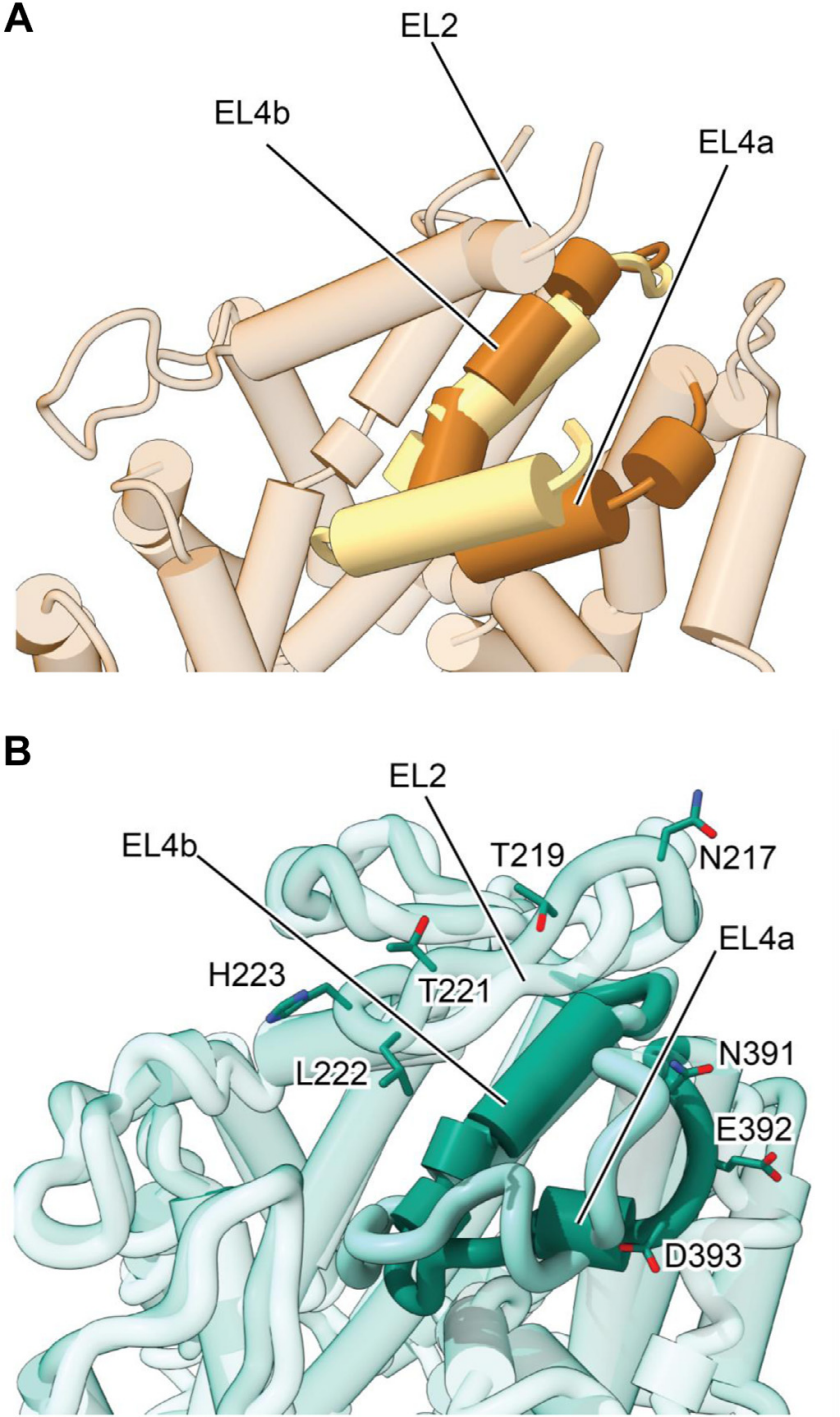
**Comparison of extracellular domain of LeuT and SERT in the inward-open and outward-open states.***A*, the outward-open state of LeuT, yellow (PDB: 3F3A) was superimposed with the inward-open state, *brown* (PBD: 3TT3). EL4 is shown in *solid colors* while the rest of the protein is transparent. *B*, the outward-open paroxetine bound state of SERT, *dark green* (PDB: 6VRH) was superimposed with the inward-open ibogaine-bound state, *light green* (PDB: 6DZZ). EL4 is shown in *solid colors* while the rest of the protein is transparent. Residues that are involved in interaction with 8B6 scFv are shown in *sticks*.

In LeuT and SERT, EL4 is composed of two α-helical segments (EL4a, EL4b), which are connected by short linkers. Comparison of the outward-open *versus* the inward-open states of LeuT (19) reveals that EL4 undergoes substantial movement upon transporter isomerization. In the outward-open state, it is exposed to solvent but, in the inward-facing state, EL4a shifts and becomes wedged between TM8 and 10, acting as a lid between scaffold domain and bundle domain, sealing off the extracellular access to the substrate permeation pathway (Fig. 10*A*). Similarly, in SERT, EL4a also forms a helical region in the outward-open state (3, 4). Residues in EL4a along with EL2 constitute a high-affinity binding site for 8B6 scFv in the outward-open state (Fig. 10*B*). In the inward-open state of SERT, EL4a undergoes a substantial conformational change causing EL4a to unwind, also causing this region to move toward TM8 and 10. These changes are also accompanied by a shift in the position of EL2, which moves “upward” toward the extracellular space. As a consequence of these rearrangements in the extracellular domain, the high-affinity binding site for 8B6 scFv is not present in the inward-open state of SERT, thus explaining why substrate induces dissociation of scFv and antiEL4 antibodies during transport. Contrary to substrate binding, binding of typical inhibitors results in a conformational trap: *i.e.*, the ligand-binding site of SERT accommodates the inhibitor, but the transporter cannot isomerize to the inward-facing state. There are, however, atypical inhibitors, which do allow for a switch to the inward-facing state. The most prominent example is ibogaine (32, 33), but there are additional atypical inhibitors (24, 34, 35). In fact, we hypothesize that there is a continuum between atypical inhibitors and partial substrates (27) and speculate that the movement of EL4 ensuing the binding event is crucial for determining, at least in part, the nature of the ligand, *i.e.*, whether it is a typical inhibitor, an atypical inhibitor, a partial, or a full substrate.

## Experimental Procedures

### Materials

Serotonin, methylphenidate, paroxetine, cOmplete protease inhibitor cocktail, buffers, salts, antibiotics, and other cell culture reagents were from Sigma-Aldrich. Fetal bovine serum (FBS) was from Biowest. [^3^H]Imipramine (specific activity 40 Ci/mmol) and [^3^H]citalopram (80 Ci/mmol) were from PerkinElmer. Glass fiber GF/C filter membranes were from Sartorius Stedim. The anti-SERT antibody (AMT-004, raised against residues 388–400 of rat SERT, affinity-purified) was from Alomone Labs. The 15B8 Fab, directed against an epitope in extracellular loop 2 (EL2), and 8B6 scFv, directed against epitopes in EL2 and EL4, were purified from Sf9 cell supernatants and bacterial lysates, respectively (3, 4).

### Electrophysiological recordings

HEK293 cells, stably expressing tetracycline-inducible wild-type GFP-tagged human SERT, were cultured in 10% fetal calf serum containing Dulbecco's Modified Eagle's Medium (DMEM). The selection pressure was maintained by adding zeocin (150 μg/ml) and blasticidin (6 μg/ml) to the medium. The cells were seeded onto 35 mm cell culture dishes coated with poly-D-lysine in 1 μg/ml tetracycline containing medium 24 h before the experiment. Cells were superfused with an external solution consisting of 140 mM NaCl, 3 mM KCl, 2.5 mM CaCl_2_, 2 mM MgCl_2_, 20 mM glucose, and 10 mM HEPES (pH = 7.4, adjusted with NaOH). The patch pipette was filled with an internal solution containing 133 mM potassium gluconate, 5.9 mM NaCl, 1 mM CaCl_2_, 0.7 mM MgCl_2_, 10 mM HEPES, and 10 mM EGTA (pH = 7.2, adjusted with KOH). In experiments requiring a high internal sodium concentration, potassium gluconate was replaced by equimolar NaCl (133 mM). Serotonin, methylphenidate, and antiSERT-EL4 antibody and antibody fragments (8B6 scFv and 15B8 Fab) were applied by using a four or eight-tube manifold combined with the Octaflow perfusion system (ALA Scientific Instruments). Electrophysiological recordings were performed in the whole-cell patch clamp configuration at room temperature using an Axopatch 200B amplifier and pClamp 10.7 software (MDS Analytical Technologies, Sunnyvale, CA). Recorded currents were filtered at 1 kHz and digitized at 5 kHz with a Digidata 1550 (MDS). Passive holding currents were subtracted, current traces were filtered by a 100 Hz digital Gaussian low-pass filter, and current amplitudes were quantified with Clampfit 10.7 software.

Membrane capacitance measurements were done as outlined previously (36).

### Radioligand-binding assay

HEK293 cells expressing wild-type human SERT were mechanically detached from the culture dish in ice-cold phosphate-buffered saline (PBS) supplemented with 0.5 mM phenylmethylsulfonyl fluoride (PMSF). Cells were centrifuged at 300*g* for 5 min at 4 °C. The pellet was resuspended in ice-cold buffer containing 20 mM HEPES.NaOH (pH 7.4), 2 mM MgCl_2_, 1 mM EDTA, 0.1 mM PMSF, and the cOmplete protease inhibitor cocktail. The cell suspension was subjected to two freeze-thaw cycles (in liquid nitrogen followed by rapid thawing) and sonicated on ice with a sonifier cell disruptor B15 (Branson Ultrasonics) by applying 12 pulses of 0.5-s duration at 50% intensity. The suspension was centrifuged at 38,000*g* for 15 min at 4 °C. The resulting pellet was resuspended in the same buffer at a protein concentration of 5–10 mg/ml. The protein concentration was determined by binding using Coomassie brilliant blue. The membranes were aliquoted, frozen in liquid nitrogen, and stored at –80 °C. Binding experiments were carried out in a final volume of 0.1 or 0.2 ml (volume adjusted to preclude radioligand depletion) of assay buffer (mM composition: 20 Tris.HCl, pH 7.4, 1 EDTA, 2 MgCl_2_, 3 KCl, 120 NaCl) containing membranes (1–2 μg), the indicated concentrations of antiSERT-EL4 antibody or of Fabs (8B6 scFv and 15B8 Fab) and of [^3^H]imipramine or of [^3^H]citalopram at 25 °C. In saturation experiments, the incubation time was 30 min. In kinetic experiments, the incubation time varied between 1 and 80 min. The antibody or the Fabs were preincubated with the membranes for 10 min at 25 °C before the addition of the radioligand. Nonspecific binding was defined in the presence of 10 μM paroxetine and was trivial (<<10% in the KD concentration range). The reaction was stopped by rapid filtration over glass fiber GF/C filter membranes, which were washed with ice-cold buffer (10 mM Tris.HCl, pH 7.4, 1 mM MgCl_2_, 120 mM NaCl). The radioactivity trapped on the filter was determined by liquid sc intillation counting at 50% efficiency.

### Modeling

Binding of 8B6 scFv to SERT was simulated by using a previously published kinetic model of the transport cycle of SERT (9). The input parameters were the kinetic rate constants for 8B6 scFv calculated from the time-course experiments. The time-dependent changes in state occupancies were evaluated by numerical integration of the resulting differential equations system using Systems Biology Toolbox (37) and MATLAB 2012a (Mathworks).

## Data availability

All data are contained within the article. All primary data are available upon request.

## Conflict of interest

E. G. is an investigator with the Howard Hughes Medical Institute. All other authors declare that they have no conflicts of interest with the contents of this article.
